# Optimal Branch Bending Angle for Korla Fragrant Pear: A Multi-Trait Physiological Trade-Off Framework

**DOI:** 10.3390/plants15020339

**Published:** 2026-01-22

**Authors:** Ablah Niyaz, Mansur Nasir, Shikui Zhang, Shaopeng Wang, Cuihui Min, Guoquan Fan, Dilraba Muhtar, Xianbiao Ma, Mirigul Tunyaz, Lihong Yao, Ruizhe Wang, Tianming He, Juan Song, Mayira Eziz

**Affiliations:** 1 Turpan Experimental Station, Xinjiang Academy of Agricultural Sciences, Turpan 838000, China; a18509960707@126.com; 2 College of Horticulture, Xinjiang Agricultural University, No. 311 Nanchang Road, Shayibak District, Urumqi 830052, China; chm699125@163.com (C.M.); 18099621925@163.com (D.M.); cuy17571087@163.com (X.M.); 18240953839@163.com (M.T.); qqing251208@163.com (L.Y.); 13602271073@163.com (R.W.); hetianming@eyou.com (T.H.); 3 National Fruit Tree Germplasm Resources Luntai Fruit Tree Resource Garden, Institute of Horticultural Crops, Xinjiang Academy of Agricultural Sciences, Korla 841600, China; zhangsk0506@xaas.ac.cn (S.Z.); wangsp@xaas.ac.cn (S.W.); 4 Department of Agricultural Science and Technology Achievement Transformation, Xinjiang Academy of Agricultural Sciences, Urumqi 830091, China; fgq2046@163.com; 5 Institute of Forestry and Horticulture, Aksu Agricultural Science and Technology Innovation Center, Aksu 843000, China; sjuan1099@163.com (J.S.); tv36839aqfd6656@163.com (M.E.)

**Keywords:** *Pyrus sinkiangensis*, branch bending angle, carbon–hormone synergy, fruit set, canopy management, high-density orchard

## Abstract

The optimal branch bending angle for *Pyrus sinkiangensis* Yü (Korla fragrant pear) remains undefined. In this study, the optimal angle was determined by integrating the phenological, nutritional, hormonal, and fruit-quality responses across a 15-day bloom window. Four branch angles (40°, 60°, 80°, and 100°) were applied to 8-year-old trees in spring 2022, and flowering dynamics, bud carbon/nitrogen status, leaf morphology/mineral content, fruiting-shoot architecture, endogenous hormones, and fruit quality were comprehensively evaluated. The 80° angle maximized the fruit set (11.77%) and bud soluble sugar content (8.84 mg/g DW), significantly outperforming the other angles (*p* < 0.05). The flowering rate peaked at 100° (7.89%) but was statistically comparable to that at 60° and 80° (*p* > 0.05); calyx removal was greatest at 60° (73.33%), with no significant difference from that at 80° (71%, *p* > 0.05). These reproductive benefits aligned with enhanced leaf source capacity—80° pulling resulted in the greatest leaf area (59.51 cm^2^), the greatest amount of chlorophyll (3.11 mg/g DW), and elevated N/Mg/Cu concentrations. Branch architecture was optimized at 80°, with the percentage of medium fruiting spurs reaching 41.1% and the xylem:phloem dry-weight ratio peaking at 1.78, indicating the development of efficient assimilate transport pathways. Hormonally, 80° triggered a distinct cascade: a transient GA_4_/GA_7_ surge (50.6 and 1.34 ng/g DW) on 28 April, followed by sustained IAA elevation (2.05 ng/g DW) and zeatin stabilization (0.27–0.29 ng/g DW) during ovary development. Consequently, the fruit quality was comprehensively improved at 80°—the single-fruit weight (110.7 g), soluble sugar content (10.08 mg/g DW), and sugar/acid ratio (17.08) were greatest, whereas the stone-cell content was lowest (0.49 mg/g DW). Principal component analysis of 57 traits confirmed 80° as the system-wide optimum (D = 0.718). These results demonstrate that an 80° bending angle synchronizes carbohydrate supply, hormone signaling, and fruit quality in Korla fragrant pear, providing a low-cost, nonchemical benchmark for precision canopy management in high-density orchards. An 80° branch-bending angle optimizes carbon-hormone synergy via a transient GA_4_/GA_7_ surge and sustained IAA-zeatin signaling, maximizing fruit set and quality in high-density Korla fragrant pear orchards.

## 1. Introduction

Branch bending is a foundational horticultural practice that regulates tree architecture, modulates source–sink relationships, and optimizes reproductive success in fruit crops [1,2]. By altering branch orientation, this technique influences light interception, auxin transport, carbohydrate partitioning, and the microclimate at the organ level, ultimately affecting flowering intensity, fruit set, and yield stability [3,4,5,6]. In high-density orchard systems—which are now globally adopted for sustainable intensification—precise canopy management is critical for balancing vegetative vigor with reproductive capacity [4]. While the practice has been successfully applied in rosaceous species such as apple (*Malus domestica*) and European pear (*Pyrus communis*) [4,7,8,9,10], optimal bending angles remain species- and genotype-specific, and their mechanistic underpinnings are largely unexplored in arid-adapted pear cultivars.

Korla fragrant pear (*Pyrus sinkiangensis* Yü) is the cornerstone of Xinjiang’s fruit industry; this fruit is cultivated across approximately 64,467 hectares and is prized for its unique aroma, crisp texture, and thin russet-free skin [11]. However, high-density plantings (4 × 2 m spacing) frequently suffer from critically low fruit set, persistent calyx end persistence, suboptimal sugar accumulation, high stone cell content, and a high incidence of coarse-skinned fruit—bottlenecks that diminish marketability and profitability [5,12]. Existing recommendations for branch angles (60–90°) derive largely from empirical observations in temperate-zone cultivars, lacking a physiology-based framework tailored to arid, high-radiation environments [5]. This leaves a critical gap between canopy manipulation and the carbon–hormone–hydraulics nexus that governs reproductive outcomes.

Recent studies have made significant progress in determining optimal bending angles for different fruit tree species. Marked cultivar-specific differences exist in apple: 90° for ‘Gala’ versus 110° for ‘Fuji’ to maximize the Carbon/Nitrogen (C/N) ratio and alleviate biennial bearing [7,8]. In walnut (*Juglans regia* ‘Qianhe 7’), 90° bending increased female flower number by 206.6% and fruit set by 14.1% [3]. For peach (*Prunus persica* ‘Luhong 618’), an 85° Y-shaped angle improved light penetration and fruit weight [4]. Wax apple (*Syzygium samarangense*) performs optimally at 65–85° [9,10]. Critically, bending ‘Fuji Mishima’ at 15 days after full bloom resulted in peak flower bud density, whereas earlier application induced ethylene inhibition [8]. Physiologically, branch bending suppresses polar auxin transport while increasing cytokinin accumulation in buds and upregulating the expression of floral identity genes such as *WUS* [6]. Branch bending significantly affects the metabolic allocation of sorbitol (the primary carbohydrate) and chlorogenic acid (the primary phenolic compound) in pear tissues, with genotype-, organ-, and time-specific responses [13,14,15]. Hormone regulation involves branch bending, which suppresses vegetative growth and promotes flowering by reducing Indole-3-acetic acid (IAA) and Gibberellin (GA) while increasing Abscisic acid (ABA) and zeatin riboside (ZR) concentrations; however, studies in apple plants further reveal that cultivar-specific maintenance of an optimal (IAA + GA + ZR)/ABA ratio is the key physiological basis for why ‘Fuji’ requires stronger bending (110°) than ‘Gala’ does (90°) [7]. Molecularly, the *LAZY* gene family mediates gravity perception via amyloplast sedimentation, and the PIN3 protein orchestrates asymmetric hormone distribution—a pathway conserved across land plants [6]. Apical control theory further explains how bending breaks assimilate suppression of lower branches, although reaction wood formation necessitates continuous angle maintenance [16]. However, despite these advances in temperate-zone cultivars, a physiology-based framework remains lacking for arid-adapted pears such as Korla fragrant pear (*Pyrus sinkiangensis* Yü), particularly for systematic analyses of the carbon–hormone–hydraulics nexus. Crucially, no study has integrated temporally resolved hormone dynamics with static morphological, nutritional, and architectural traits to define a system-level optimum for this economically vital cultivar.

To address this gap, we conducted the first comprehensive, multitrait evaluation of four branch angles (40°, 60°, 80°, and 100°) in an 8-year-old orchard, integrating 57 phenotypic, nutritional, and hormonal traits measured across critical developmental stages—from flowering through fruit set to harvest. This framework captures the carbon–hormone–hydraulics nexus, with a 15-day hormone monitoring window during blooms. We hypothesized that branch angle mediates carbon assimilation, nutrient allocation, and hormone dynamics, creating angle-specific trade-offs between reproductive physiology and vegetative stress. Using Liquid Chromatography–Tandem Mass Spectrometry (LC–MS/MS)-based hormone profiling, principal component analysis (PCA), and a novel multitrait physiological trade-off framework, we quantified angle-specific effects on reproductive development, identified key trait covariances, and developed a predictive framework for precision canopy management. This work provides a mechanistic template for the transformation of empirical horticultural practices into predictive science, with direct implications for the sustainable intensification of rosaceous fruit crops in water-limited environments.

## 2. Materials and Methods

### 2.1. Plant Material and Experimental Site

The trial was conducted at the Luntai Fruit Tree Resource Nursery (41.8° N, 84.1° E, 962 m a.s.l.), Bayingolin, Xinjiang, China. Eight-year-old ‘Korla fragrant pear’ (*Pyrus sinkiangensis* Yü) trees grafted onto Duli (*Pyrus betulifolia* Bunge) rootstock with Zhongai 1 (ZA1) interstock (Duli/ZA1) (4 × 2 m spacing) were used. The region has a temperate continental arid climate (annual mean 10.6 °C, 52 mm of precipitation, and 188 frost-free days). Twelve uniform trees were selected and arranged in a randomized complete block design with three replicates per treatment (n = 3 trees). The 40° treatment served as the control, representing the natural branch angle of unmanipulated scaffold branches in 8-year-old ‘Korla fragrant pear’ trees. Three additional bending angles (60°, 80°, and 100° from the vertical) were applied to the main scaffold branches. Branch angles were set using nylon straps (5 cm width) with cotton padding to prevent bark injury, looped at 30 cm intervals, and secured to ground anchors. Angles were measured with a digital protractor (Suunto PM-5/360 PC, Vantaa, Finland; deviation ≤ ±3°). Straps were inspected biweekly and removed after 2–3 months once angles stabilized. Treatments were applied in early June 2021 to allow a complete annual cycle of carbohydrate accumulation and vascular remodeling before the 2022 bloom period, as suggested by the time-specific responses reported in previous studies [13,14,15].

### 2.2. Flowering Phenology and Fruit Set

Flowering stages (initial, full, and petal-fall) were recorded daily. Flowering rate (%) = flower buds/total buds × 100. After the plants were manually thinned to a uniform flower load, the following formula was used: Fruit set rate (%) = (fruits remaining 10 d after the first physiological drop/thinning flowers) × 100. The calyx removal rate (%) = calyx-shed fruits/total fruits × 100 was scored at the end of the second physiological drop.

### 2.3. Bud Nutrients

At the green-cluster stage, terminal buds of short shoots and axillary buds of long shoots were collected, immediately frozen in liquid nitrogen (N_2_), freeze-dried, and stored at –80°. Soluble sugars were determined by the anthrone-sulfuric colorimetric method, and total nitrogen was measured by the standard Kjeldahl acid-digestion method [7]. Each treatment had five independent replicates.

### 2.4. Leaf Morphology, Chlorophyll and Mineral Elements

In September 2022, 100 healthy leaves per treatment were sampled. The leaf length, width, fresh and dry weights, and area (LI-3100C, LI-COR, Lincoln, NE, USA) were recorded. Chlorophyll a, b and total were extracted in 80% acetone (analytical grade, Macklin, Shanghai, China) and quantified spectrophotometrically; Soil Plant Analyzer Development (SPAD) values were obtained with a SPAD-502 Plus chlorophyll meter (Konica Minolta, Chiyoda-ku, Tokyo, Japan). Minerals (including nitrogen (N), calcium (Ca), magnesium (Mg), iron (Fe), zinc (Zn), and copper (Cu) were determined by inductively coupled plasma optical emission spectrometry (ICP–OES; PerkinElmer Optima 8300, Waltham, MA, USA) after HNO_3_–HClO_4_ (trace metal grade, Merck, Darmstadt, Germany) digestion following the methods of Masson et al. [17]. Element selection rationale: N, Mg and Cu were prioritized for their roles in chlorophyll and electron transport; Ca, Fe and Zn were included as key cofactors. K and P were omitted due to high phloem mobility and limited short-term response to branch-angle manipulation [13,14,15,18].

### 2.5. Branch Growth and Water Status

After shoot cessation, 20 branches per treatment were classified into short (<5 cm), medium (5–15 cm) and long (>15 cm) fruiting spurs; length and basal diameter were recorded. Five one-year-old branches per treatment were segmented into xylem and phloem; fresh and dry weights were used to calculate the water content (%). The total nitrogen in the xylem and phloem tissues was determined by the Kjeldahl method [19].

### 2.6. Fruit Quality Assessment

At harvest, 30 fruits per treatment were randomly collected. External indices, including single-fruit weight, shape index, pedicel length and diameter, surface finish, color and russeting index and internal indices, including flesh firmness, soluble solids, soluble sugar, titratable acidity, vitamin C and stone-cell content, were determined as described previously [12].

### 2.7. Endogenous Hormone Profiling During the Young-Fruit Stage

Young fruits were sampled every 3 days from 19 April (the second day after full bloom in the 40° and 60° plots and the third day in the 80° and 100° plots) until 4 May (when calyx abscission was >95%). Ten fruits per replicate were pooled, immediately frozen in liquid N_2_ and stored at −80 °C. Endogenous hormones (IAA, ABA, gibberellin A_1_ (GA_1_), gibberellin A_3_ (GA_3_), gibberellin A_4_ (GA_4_), gibberellin A_7_ (GA_7_), salicylic acid (SA), jasmonic acid (JA), cis-zeatin (CZ), and trans-zeatin (TZ)) were extracted and purified by a modified method [20] and analyzed by LC–MS/MS (Shimadzu LC-30AD, Kyoto, Japan; coupled with AB Sciex QTRAP 6500+, Framingham, MA, USA).

### 2.8. Statistical Analysis

All the data were analyzed in IBM SPSS Statistics ver. 27 (June 2019, IBM Corporation, Armonk, NY, USA). One-way Analysis of Variance (ANOVA) followed by Duncan’s test was applied when *p* < 0.05. Correlation heatmaps and radar charts were generated with OriginPro 2025 ver. 10.2.0.188 (OriginLab Corporation, Northampton, MA, USA).

#### Principal Component Analysis (PCA) and Comprehensive Evaluation

To integrate the 57 phenotypic and physiological traits into a single ranking metric, PCA was performed. Three principal components (PC1–PC3) were extracted and rotated using the varimax method. The component scores were transformed into comprehensive indices (CI_1_, CI_2_, CI_3_) and combined into a final evaluation score (D) using the membership function approach. Higher D values indicate superior overall performance. All PCA computations were carried out in OriginPro 2025.

## 3. Results

To systematically evaluate how the branch-bending angle regulates reproductive development and whole-plant physiology in *Pyrus sinkiangensis*, we quantified (i) flowering phenology, (ii) flowering rate, fruit set and calyx removal, (iii) bud soluble sugar and nitrogen, (iv) leaf fresh/dry mass, area and chlorophyll, (v) leaf mineral profile, (vi) fruiting-shoot typology and dimensions, (vii) xylem vs. phloem water content, (viii) branch nitrogen and dry-mass partitioning, (ix) external and internal fruit quality, (x) young-fruit endogenous hormone dynamics, and (xi) trait intercorrelations via heat-map and radar analyses. The resulting datasets were integrated by univariate statistics and principal component analysis to identify the canopy configuration best suited for high-density pear orchards.

### 3.1. Flowering Phenology and Reproductive Performance

#### 3.1.1. Impact of Branch Bending Angle on the Flowering Phenology of Korla Fragrant Pear

**Figure 1:**
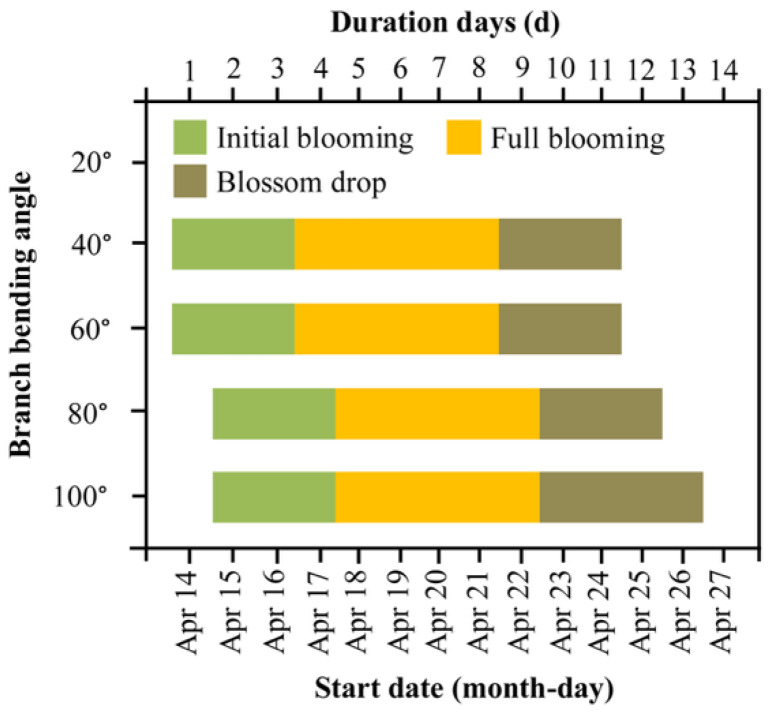
Flowering phenology of Korla fragrant pear at four branch-bending angles. Initial bloom (5% open flowers), full bloom (25–75% open flowers) and petal fall (75–95% petals dropped) were monitored daily from 14 to 26 April 2022. The data are presented as the duration (days) of each phase.

We first tested whether the bending angle shifts the timing of key flowering events. Regardless of treatment, bud burst occurred uniformly on 5 April, while full bloom peaked on 14–15 April, and petal fall was completed by 24–26 April, resulting in an overall flowering window of ca. 11 days (Figure 1). Only marginal angle effects were detected: initial flowering was delayed by 1 day under 80° and 100° relative to 40° and 60°, and the blossom-drop phase of 100° lasted one day longer than that of 80° (*p* < 0.05). Thus, larger bending angles slightly delayed anthesis and extended the blooming period without advancing or shortening the entire flowering season.

#### 3.1.2. Impact of Branch Bending Angle on the Flowering Rate and Fruit Set of Korla Fragrant Pear

**Figure 2:**
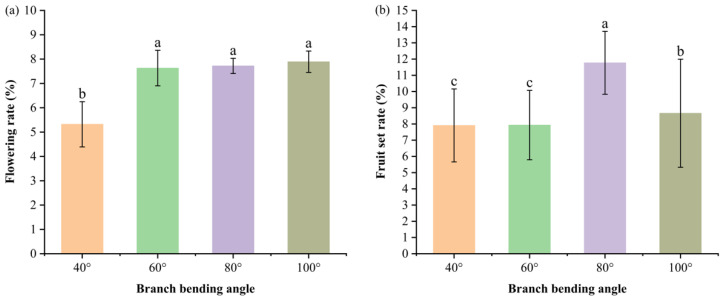
Flowering rates (**a**) and fruit set rates (**b**) of Korla fragrant pears with different branch-bending angles. The data are presented as the means ± SEs; n = 5 branches per angle. Different letters above the bars denote significant differences (Duncan’s test, *p* < 0.05).

The flowering rate (flowering buds/total buds) and fruit set (retained fruits after the first physiological drop/thinned flowers) were recorded from April–May 2022. The 40° angle resulted in the lowest flowering rate (*p* < 0.05 versus all the other angles); the values did not differ between 60–100°, where 100° reached 7.89%. Fruit set peaked at 80° (11.77%), followed by 100° (8.66%), and both exceeded 40° and 60° (*p* < 0.05) (Figure 2). Thus, 80° pulling optimized the fruit set, whereas the flowering rate was suppressed only at 40°.

### 3.2. Impact of Branch Bending Angle on Leaf Morphology, Chlorophyll Content and Mineral Composition of Korla Fragrant Pear

To test whether branch orientation alters source-leaf morphology, photosynthetic potential and mineral supply capacity, we determined single-leaf fresh mass, dry mass, area, linear dimensions, chlorophyll pigments and mineral element concentrations across the four bending angles.

**Figure 3:**
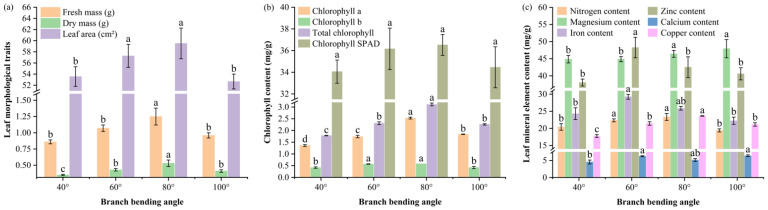
Leaf morphology, chlorophyll content and mineral element concentrations of Korla fragrant pear at four bending angles. (**a**) Single-leaf fresh mass, dry mass and area; the *Y*-axis is broken between 1.6 and 51 to better visualize differences. (**b**) Chlorophyll a, chlorophyll b, total chlorophyll and total chlorophyll SPAD; the *Y*-axis is broken between 3.2 and 31.5 mg/g. (**c**) Nitrogen, magnesium, iron, zinc, calcium and copper; the *Y*-axis is broken between 7.5 and 14 mg/g and between 31 and 36.5 mg/g. Mean ± SE, n = 5 leaves per angle. Different letters indicate significant differences (Duncan’s test, *p* < 0.05).

The leaf morphology data revealed that moderate bending (60–80°) significantly increased all the growth metrics: 80° resulted in the greatest single-leaf fresh mass (1.25 g), the heaviest dry mass (0.53 g) and the greatest leaf area (59.51 cm^2^), all of which were significantly greater than those at 40° and 100° (*p* ≤ 0.05), whereas 60° ranked second and 40°/100° presented the smallest values (Figure 3a).

The chlorophyll content mirrored the morphological response; at 80°, the chlorophyll a, chlorophyll b and total chlorophyll (a + b) contents reached 2.53, 0.58 and 3.11 mg g^−1^, respectively—significantly higher than those in all the other treatments (*p* ≤ 0.05), whereas the SPAD values showed the same numerical trend (36.52 at 80°), but the values did not significantly differ (*p* > 0.05), indicating a visually greener yet statistically comparable photosynthetic unit under moderate bending (Figure 3b).

Leaf mineral profiling (Figure 3c) revealed that the 80° pulling treatment modified the macronutrient and micronutrient concentrations: the concentration of nitrogen reached 23.36 mg/g, which was significantly greater than that at 40° (20.42 mg/g) and 100° (19.42 mg/g) (*p* ≤ 0.05) but not different from that at 60° (22.36 mg/g) (*p* > 0.05); the concentration of magnesium peaked at 46.43 mg/g, which was significantly greater than that at 40° (44.86 mg/g) and 60° (44.88 mg g^−1^) (*p* ≤ 0.05) but was comparable to that at 100° (47.94 mg/g) (*p* > 0.05); the concentration of copper was greatest at 23.64 mg g^−1^, which exceeded all the other angles (*p* ≤ 0.05); the concentrations of iron (25.87 mg/g) and calcium (5.14 mg/g) did not differ significantly from those at any angle (*p* > 0.05); and the concentration of zinc (42.53 mg/g) was significantly lower than that at 60° (48.27 mg/g) (*p* ≤ 0.05) but not significantly different from that at 40° (38.04 mg/g) and 100° (40.59 mg/g) (*p* > 0.05). Overall, the 80° pulling treatment elevated leaf N, Mg and Cu availability, increasing the source capacity for fruit development. Notably, the concurrent increase in Mg (46.43 mg/g) and chlorophyll content at 80° aligns with Mg’s central role in chlorophyll porphyrin rings, while elevated Cu (23.64 mg/g) may enhance photosynthetic electron transport via plastocyanin, collectively supporting the observed increase in leaf photochemical capacity.

### 3.3. Impact of Branch Bending Angle on Fruiting Shoot Typology

To evaluate whether branch angle reallocates assimilates by modifying fruiting architecture, we classified spur types and measured the corresponding branch dimensions across the four bending angles.

**Figure 4:**
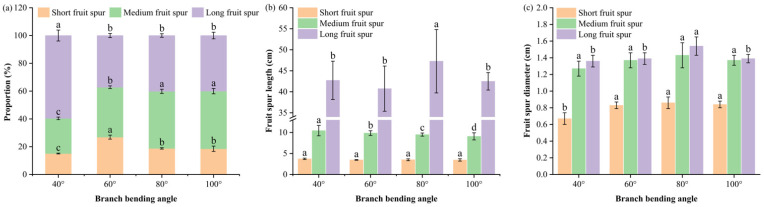
Fruiting-shoot typology and branch-dimensional traits of Korla fragrant pear under four branch-bending angles: (**a**) proportions of short (< 5 cm), medium (5–15 cm) and long (> 15 cm) fruiting spurs. (**b**) Fruit spur length; the *Y*-axis is broken between 13 and 34 cm to better visualize differences. (**c**) Fruit spur diameter. Mean ± SE, n = 5 branches per angle. Different letters indicate significant differences (Duncan’s test, *p* < 0.05).

Fruiting-shoot composition (Figure 4a) revealed that pulling ≥ 60° significantly mitigated shoot vigor and rebalanced vegetative vs. reproductive competition. Compared with the 40° treatments, the 60°, 80° and 100° treatments collectively increased the combined share of short plus medium spurs and reduced the proportion of long spurs. The short-spur percentage was greatest at 60° (26.9%), followed by 80° (18.8%) and 100° (18.5%), which were significantly greater than 40° (15.1%) (*p* ≤ 0.05). The medium-spur percentage peaked at 80° and 100° (41.1% and 41.5%, respectively), significantly exceeding 40° (25.3%) and 60° (35.9%) (*p* ≤ 0.05). The long-spur proportion at 60°, 80° and 100° (~37–40%) was significantly lower than that at 40° (59.6%) (*p* ≤ 0.05), with no difference among the three larger angles (*p* > 0.05).

The branch dimension traits indicated that short-spur length did not differ significantly among the angles (*p* > 0.05), whereas the medium-spur length at 80° (9.50 cm) was significantly shorter than that in the other three treatments (*p* ≤ 0.05), and the long-spur length at 80° (47.3 cm) was significantly greater than that at 40°, 60° and 100° (*p* ≤ 0.05) (Figure 4b). The short-spur basal diameter at 60°, 80° and 100° (≥ 0.83 cm) was significantly greater than that at 40° (0.67 cm) (*p* ≤ 0.05). The medium-spur diameter peaked at 80° (1.43 cm) but was not significantly different from the other angles (*p* > 0.05). The long-spur diameter was greatest in the 80° treatment (1.54 cm), significantly exceeding that in all the other treatments (*p* ≤ 0.05) (Figure 4c).

Thus, the 80° pulling treatment restructured the canopy into shorter but thicker fruiting spurs, creating more efficient “unloading ports” that reconcile vegetative vigor with reproductive sink strength.

#### Impact of Branch Bending Angle on Branch Water Status and Nitrogen Reserve

To test whether the bending angle affects the water–nitrogen coupling that underpins subsequent fruit development, we quantified the xylem/phloem water content, tissue ratio and total nitrogen across branch segments.

**Figure 5:**
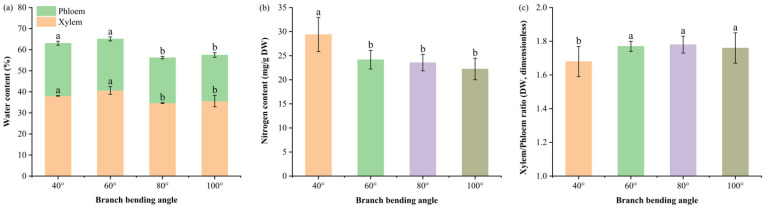
Water content in the xylem and phloem, branch nitrogen concentration and dry weight ratio of the xylem to phloem of Korla fragrant pear under four branch-bending angles: (**a**) Stacked water content of the xylem and phloem; letters above segments indicate significant differences within the same tissue (Duncan’s test, *p* < 0.05). Total water content: 40° = 63.1%, 60° = 65.1%, 80° = 56.2%, 100° = 57.2%; letters in parentheses denote total-water comparison. (**b**) Total nitrogen concentration in the whole branch. (**c**) Xylem-to-phloem dry-weight ratio. Mean ± SE, n = 3 branches per angle. Different letters indicate significant differences (Duncan’s test, *p* < 0.05).

Branch water partitioning (Figure 5a) revealed that the 80° pulling treatment significantly decreased the total branch water content to 56.2% compared with 40° (63.1%) and 60° (65.1%) (*p* ≤ 0.05), whereas no difference was detected compared with 100° (57.2%) (*p* > 0.05). The phloem water content under 80° (21.5%) was also significantly lower than that under 40° and 60° (*p* ≤ 0.05) but remained comparable to that under 100° (*p* > 0.05).

The xylem-to-phloem dry weight ratio (Figure 5b) peaked at 1.78 under 80°, which was significantly greater than 40° (1.68) (*p* ≤ 0.05) but did not differ from 60° (1.77) and 100° (1.76) (*p* > 0.05). This ratio, combined with reduced total branch water content at 80° (Figure 5a) and elevated leaf nitrogen (Figure 3c), suggests a hydraulic architecture that balances drought-tolerance with efficient nutrient transport.

The tissue nitrogen reserves (Figure 5c) were highest for total branch N at 40° (29.40 ± 3.56 mg g^−1^ DW), which were significantly greater than those at 60°, 80° and 100° (22–24 mg g^−1^ DW) (*p* ≤ 0.05), with no significant differences among the latter three (*p* > 0.05). Thus, 40° provided the greatest nitrogen reservoir within the branch, while larger bending angles maintained a lower but statistically equivalent N pool.

Taken together, 80° bending establishes a water-restricted yet nitrogen-adequate branch framework that prevents hydraulic overload while supplying sufficient mineral nutrients, creating a physiological precondition for premium fruit formation.

### 3.4. Impact of Branch Bending Angle on the Nutrient Contents of Korla Fragrant Pear Buds

To test whether the superior fruit set and calyx removal observed at 80° and 60°, respectively, stem from enhanced carbon/nitrogen build-up in the buds, we determined the soluble sugar and total nitrogen concentrations at the green-cluster stage.

**Figure 6:**
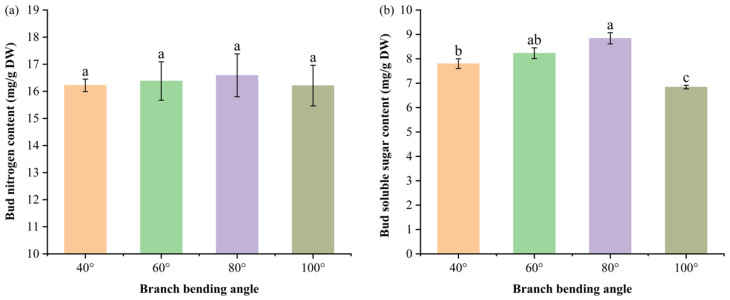
Bud nitrogen and soluble sugar contents of Korla fragrant pear at four branch-bending angles: (**a**) nitrogen content; (**b**) soluble sugar content. Mean ± SE, n = 5 bud replicates per angle. Different letters indicate significant differences (Duncan’s test, *p* < 0.05).

Total nitrogen was highest at 80° (16.59 mg/g DW) but did not differ significantly among the angles (*p* > 0.05) (Figure 6a). The soluble sugar content (Figure 6b) peaked at 80° (8.84 mg/g DW), which was significantly greater than that at 40° (7.80 mg/g DW) and 100° (6.84 mg/g DW) (*p* ≤ 0.05) and comparable to that at 60° (8.23 mg/g DW) (*p* > 0.05). Consequently, the C: N ratio was maximized under 80°, favoring flower bud differentiation and subsequent fruit set.

Taken together, moderate pulling (60–80°) enhances bud carbon status while maintaining adequate nitrogen, providing a biochemical basis for the greater fruit set observed at 80°.

### 3.5. Impact of Branch Bending Angle on Endogenous Hormone Dynamics in Young Fruits at Key Stages of Calyx Abscission and the First Physiological Fruit Set of Korla Fragrant Pear

To elucidate the hormonal mechanisms underlying angle-dependent fruit set, we monitored ten endogenous hormones in young Duli/ZA1 from 19 April to 4 May—a critical window spanning calyx abscission and the first period of physiological fruit drop. Hormone profiling was performed using LC–MS/MS to explore potential associations between branch bending angles and the patterns of calyx shedding and fruit set.

**Figure 7:**
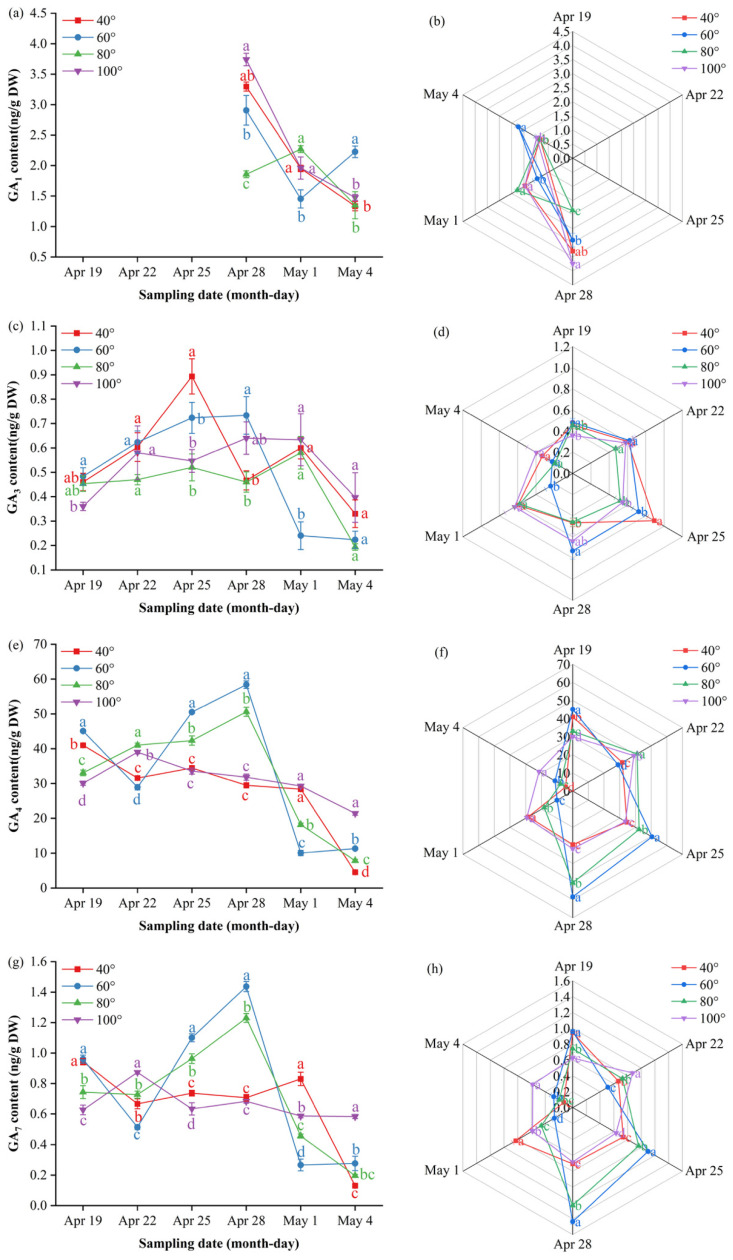
Dynamic GA content and multi-index radar profile comparison: (**a**) changes in the GA_1_ content of Korla fragrant pear young fruits at key sampling stages under different branch bending angles; (**b**) GA_1_ multi-index radar profile; (**c**) changes in the GA_3_ content; (**d**) GA_3_ multi-index radar profile; (**e**) changes in the GA_4_ content; (**f**) GA_4_ multi-index radar profile; (**g**) changes in the GA_7_ content; (**h**) GA_7_ multi-index radar profile. Mean ± SE, n = 3 biological replicates. Different letters indicate significant differences among the angles on the same sampling date (Duncan’s test, *p* < 0.05).

Gibberellin A_1_ (GA_1_) remained below the detection limit until 28 April, when it surged transiently: 100° peaked at 3.74 ng/g DW, significantly higher than the other angles (*p* ≤ 0.05), and then fell rapidly toward a low plateau by 4 May. On 1 May, 80° rebounded to 2.27 ng/g DW, surpassing 60° (*p* ≤ 0.05), whereas 60° ultimately took the lead at 2.23 ng/g DW on 4 May (*p* ≤ 0.05) (Figure 7a, Table S1). The radar profile (Figure 7b) illustrates this handover: 100° dominates the late-April apex, 80° decreases to the bottom segment, and 60° emerges as the final peak.

Gibberellin A_3_ (GA_3_) followed a “low–spike–decline” trajectory, with only a transient elevation at 40° on 25 April before it returned to baseline levels across all the angles (Figure 7c, Table S1). The multi-index radar profile (Figure 7d) visualizes these dynamics: 80° presents a low-and-flat band throughout; 60° follows an “early-high, then-fading” arc; and 40° and 100° occupy mid-peak sectors on 25 and 28 April, respectively, with 100° showing a “low-to-high” rebound trajectory and 40° maintaining elevated levels after its peak.

Gibberellin A_4_ (GA_4_) exhibited a “rise–peak–decline” pattern at 60° and 80°, peaking on 28 April at 58.4 ng/g DW and 50.6 ng/g DW, respectively—both significantly higher than 40° and 100° (*p* ≤ 0.05)—before it reached baseline. In contrast, 40° and 100° showed a steady downward trend after early April; only 100° displayed a minor late rebound to 21.5 ng/g DW on 4 May (Figure 7e, Table S1). The radar profile (Figure 7f) mirrors this divergence: 60°/80° occupies the late-April apex sector, whereas 40° remains low and 100° traces a gentle “low-to-high” tail. and 100° traces a gentle “low-to-high” tail.

Gibberellin A_7_ (GA_7_) showed a “rise–peak–decline” pattern at 60° and 80°, peaking on 28 April at 1.44 ng/g DW and 1.34 ng/g DW, respectively—both significantly higher than 40° and 100° (*p* ≤ 0.05)—before it returned to baseline. In contrast, 40° remained essentially flat throughout, whereas 100° remained low until a sharp decrease on 4 May (Figure 7g, Table S1). The radar profile (Figure 7h) captures this divergence: 60°/80° dominates the late-April apex sector, 40° traces a low horizontal band, and 100° displays a terminal “cliff” descent.

**Figure 8:**
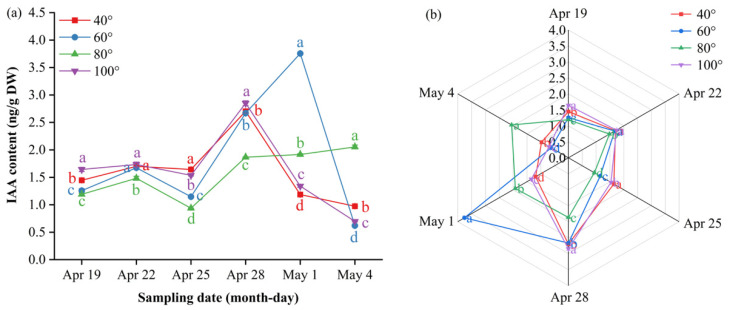
Dynamic IAA content and multi-index radar profile comparison. (**a**) Changes in the IAA content of Korla fragrant pear young fruits at key sampling stages under different branch bending angles; (**b**) radar profile of multiple indices for the four angles. Mean ± SE, n = 3 biological replicates. Different letters indicate significant differences among the angles on the same sampling date (Duncan’s test, *p* < 0.05).

Indole-3-acetic acid (IAA) showed a “flat–surge–decline” trend at 40°, 60° and 100°, whereas 80° followed a “flat–surge–plateau” pattern (Figure 8a, Table S1). Before 28 April, 80° and 60° were significantly lower than 40° and 100° (*p* ≤ 0.05); thereafter, 80° continued to rise and plateaued at 2.05 ng/g DW on 4 May, remaining significantly higher than all the other angles at the final two samplings (*p* ≤ 0.05). In contrast, 40°, 60° and 100° peaked on approximately 28 April or 1 May and decreased markedly by 4 May; the minimum value (0.62 ng/g DW) was recorded at 60°. The radar plots (Figure 8b) mirror the late-phase dominance of 80°.

**Figure 9:**
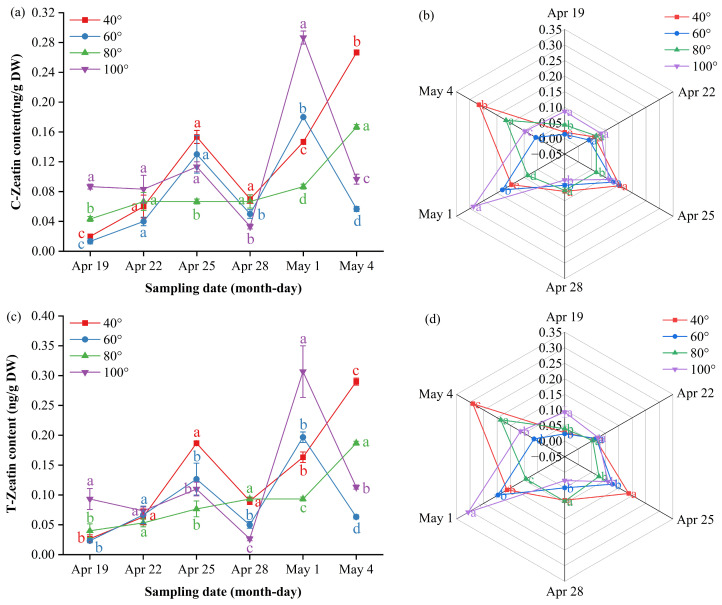
Dynamic cytokinin content and multi-index radar profile comparison: (**a**) changes in the cis-zeatin content of Korla fragrant young pear fruits at key sampling stages under different branch bending angles; (**b**) cis-zeatin multi-index radar profile; (**c**) changes in the trans-zeatin content; (**d**) trans-zeatin multi-index radar profile. Mean ± SE, n = 3 biological replicates. Different letters indicate significant differences among the angles on the same sampling date (Duncan’s test, *p* < 0.05).

Cis- and trans-zeatin in the 80° treatment followed a “slow-rise–steep-rise” two-phase pattern: levels remained low to moderate from 19 to 28 April but then increased rapidly to simultaneous peaks on 4 May (CZ 0.27 ng/g DW; TZ 0.29 ng/g DW), which were significantly greater than 40° and 60° (*p* ≤ 0.05) but still lower than 100°. The 40° angle displayed an overall upward trend, with two distinct peaks on 25 April and 4 May, whereas 60° and 100° peaked on 1 May and then declined (Figure 9a,c, Table S1). The radar profiles (Figure 9b,d) show 100° as the outermost sector on 1 May, 40° as it exhibited dual protrusions on 25 April and 4 May, and 80° as it expanded only on 4 May, confirming the angle × time interaction.

**Figure 10:**
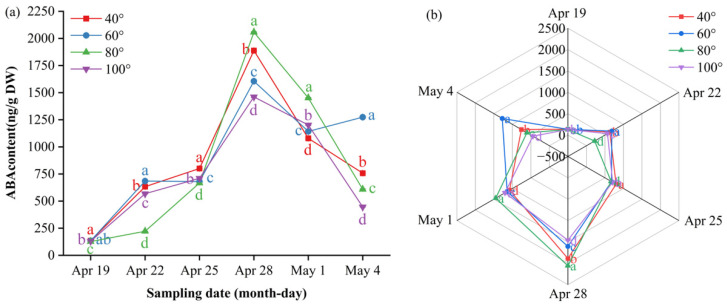
Dynamic ABA content and multi-index radar profile comparison: (**a**) changes in the ABA content of Korla fragrant pear young fruits at key sampling stages under different branch bending angles; (**b**) ABA multi-index radar profile. Mean ± SE, n = 3 biological replicates. Different letters indicate significant differences among the angles on the same sampling date (Duncan’s test, *p* < 0.05).

Abscisic acid (ABA) exhibited a “surge–decline” pattern. Before 28 April, 80° remained in the inner ring of the radar plot, with concentrations significantly lower than those reached later (*p* ≤ 0.05). The values then increased, peaking on 28 April at 2058 ng/g DW at 80° and significantly higher than those at 60°, 100° and 40° (*p* ≤ 0.05); this dominance persisted on 1 May (1451 ng/g DW). Thereafter, the levels decreased; on 4 May, they ranked 60° (1274 ng/g DW) > 40° (757 ng/g DW) > 80° (609 ng/g DW) > 100° (446 ng/g DW) (*p* ≤ 0.05) (Figure 10a, Table S1). Radar plots (Figure 10b) show 80° at the outermost arc on 28 April and 1 May, whereas 60° expands to the periphery only on 4 May, illustrating the sequential shift in ABA dominance during calyx abscission.

**Figure 11:**
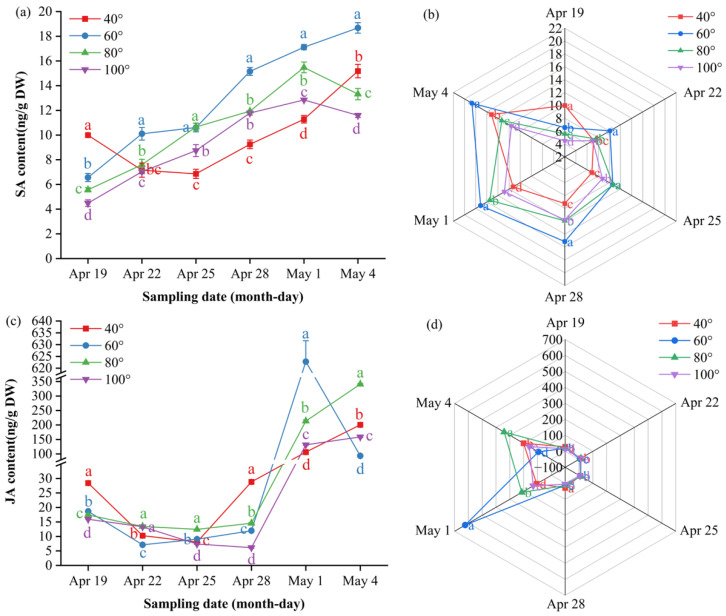
Dynamic salicylic acid (**a**,**b**) and jasmonic acid (**c**,**d**) contents of Korla fragrant pear young fruits at key sampling stages under different branch-bending angles. (**a**,**c**) Time-course changes; (**b**,**d**) multi-index radar profiles. Mean ± SE, n = 3 biological replicates. Different letters indicate significant differences among the angles on the same sampling date (Duncan’s test, *p* < 0.05).

Salicylic acid (SA) dynamics split into two clear tracks. The high-angle group (80°, 100°) climbed steadily until 1 May, peaking simultaneously at 15.48 ng/g DW (80°) and 12.85 ng/g DW (100°), each significantly higher than 40° (*p* ≤ 0.05), and then declined sharply; by 4 May, both had fallen to their respective minima—13.32 ng/g DW for 80° and 11.59 ng/g DW for 100°—which were significantly lower than 40° and 60° (*p* ≤ 0.05). The low-angle group (40°, 60°) showed divergent paths: 40° bottomed out on 25 April (6.86 ng/g DW, *p* ≤ 0.05) before rising continuously to 15.18 ng/g DW on 4 May, whereas 60° increased almost linearly to the overall maximum of 18.68 ng/g DW on 4 May (*p* ≤ 0.05). Consequently, 60° maintained the highest SA level across the window, with 80° holding second place until 1 May (Figure 11a, Table S1). The radar plots (Figure 11b) mirror this switch: from 22 April onward, 60° and 80° occupy the outer rings, whereas 40° and 100° stay closer to the center; on 1 May, the 80° sector reaches its maximum outward extension, and by 4 May, only 60° expands to the periphery, visually underlining the angle- and time-specific SA burst during calyx abscission.

Jasmonic acid (JA) displayed a “flat–surge” pattern at 40°, 80° and 100° and a “flat–surge–decline” pattern at 60°. Basal concentrations ≤ 30 ng/g DW were maintained until 28 April, after which all the angles rose rapidly. On 1 May, the peak appeared at 60° at 622.8 ng/g DW, with 80° in second place at 213.6 ng/g DW, which were significantly greater than those at 100° (131.6 ng/g DW) and 40° (106.6 ng/g DW) (*p* ≤ 0.05). Thereafter, 60° declined sharply, while the remaining angles continued to increase; by 4 May, the ranking was 80° (341.0 ng/g DW) > 40° (200.7 ng/g DW) > 100° (159.1 ng/g DW) > 60° (93.5 ng/g DW), with significant differences between each step (*p* ≤ 0.05) (Figure 11c, Table S1). Overall, 80° maintained a consistently high level throughout the window. Radar plots (Figure 11d) show 80° occupying the outermost arc from 22 April onward, whereas 40° and 100° remain closer to the center, visually confirming the angle- and time-dependent JA burst during calyx abscission.

#### 3.5.1. April 28 Served as the Pivotal Hormonal Switch Point

Before this date, low levels of IAA and zeatin (cis-/trans-zeatin) permitted calyx abscission, whereas simultaneous surges of GA_4_, GA_7_, ABA, SA and JA initiated separation. After 28 April, the levels of these hormones decreased rapidly, whereas those of IAA and zeatin increased steadily and peaked on 1–4 May; this “second wave” reinforced ovary IAA–zeatin signaling, antagonizing ABA-induced abscission reactivation and thus reducing the first degree of physiological fruit drop. Concentrations >0.30 ng g^−1^ DW risk reactivating cell division, promoting fruit enlargement and increasing persistent calyx fruits. The 80° bending angle precisely maintained the zeatin concentration at 0.27–0.29 ng g^−1^ DW, combined with high levels of soluble sugars and IAA, minimized calyx retention and maximized fruit set (11.8%), providing a practical physiological basis for high-density Korla fragrant pear orchards.

#### 3.5.2. Impact of Branch Bending Angle on Consumer-Grade Fruit Quality

**Figure 12:**
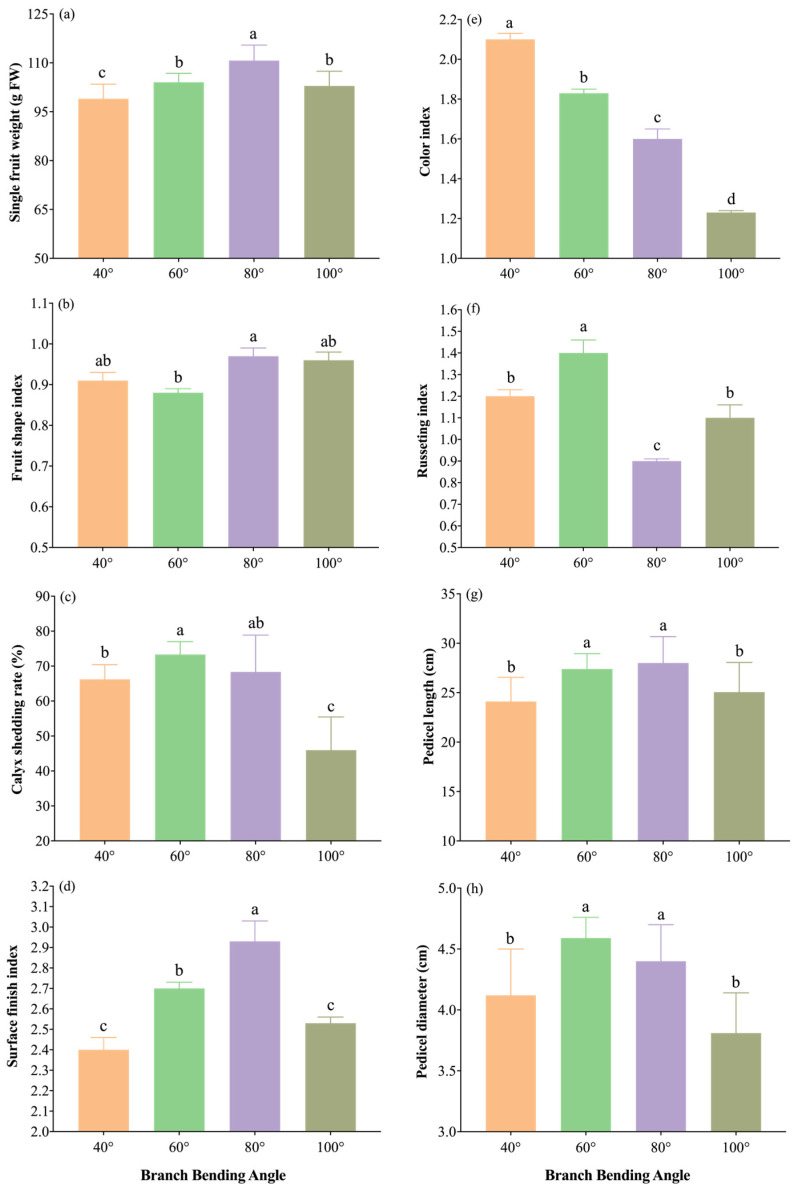
External fruit quality traits of Korla fragrant pear under four branch-bending angles: (**a**) single-fruit weight, (**b**) fruit shape index, (**c**) calyx shedding rate, (**d**) surface finish index, (**e**) color index, (**f**) russeting index, (**g**) pedicel length, and (**h**) pedicel diameter. Mean ± SE, n = 30 fruit per angle. Different letters indicate significant differences (Duncan’s test, *p* < 0.05).

**Figure 13:**
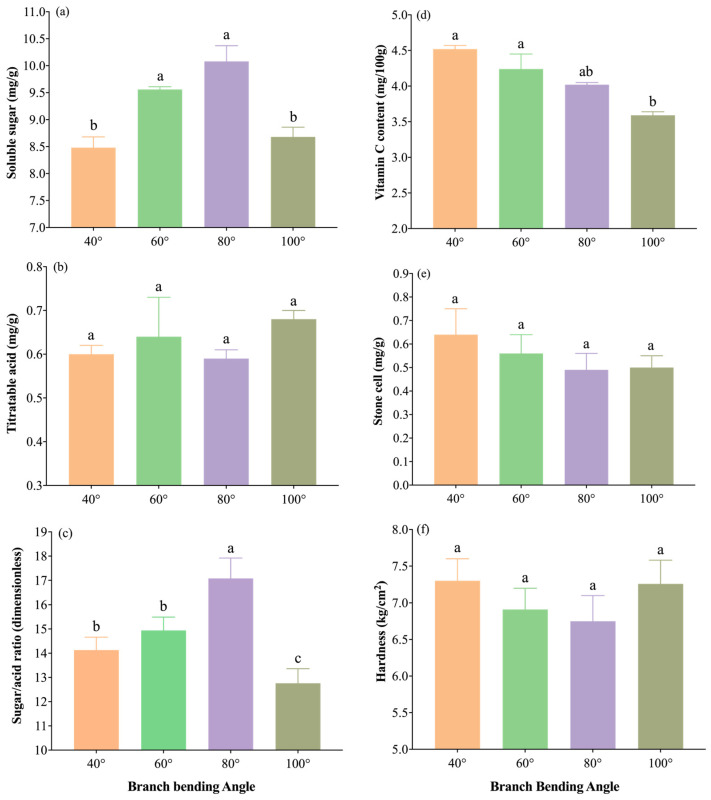
Internal fruit quality traits of Korla fragrant pear under four branch-bending angles: (**a**) sugar/acid ratio, (**b**) soluble sugar content, (**c**) titratable acid content, (**d**) vitamin C content, (**e**) stone-cell content, and (**f**) hardness. Mean ± SE, n = 30 fruit per angle. Different letters indicate significant differences (Duncan’s test, *p* < 0.05).

Harvest measurements revealed that the bending angle significantly modified both external and internal quality traits (Figure 12 and Figure 13).

The external quality traits are summarized in Figure 12a–h. The 80° treatment produced the heaviest fruit (110.7 g), significantly outweighing all the other angles (*p* ≤ 0.05). Calyx removal under 80° (71%) was statistically comparable to the maximum in 60° (73%, *p* > 0.05), ensuring a smooth shoulder. The shape index peaked at 0.97 under 80°, whereas the peel smoothness (cleaning index of 2.93) was the highest, and the color uniformity (rust index of 0.90) was intermediate—higher than 100° but lower than 40° and 60°—yielding the most attractive overall finish. Additionally, 80° yielded the longest pedicel (28.0 cm) and thickest stalk base (4.40 mm), which were significantly greater than those of 40° and 100° (*p* ≤ 0.05). Overall, 80° delivers the largest, cleanest and most uniformly finished fruit, providing a clear market advantage for high-density Korla fragrant pear production.

The internal quality indices are shown in Figure 13a–f. The 80° treatment resulted in the highest soluble-sugar content (10.08 mg g^−1^), significantly outperforming 40° and 100° (*p* ≤ 0.05) but being comparable to 60° (*p* > 0.05); it also resulted in the highest sugar/acid ratio (17.08), which was significantly greater than that at all the other angles (*p* ≤ 0.05). Titratable acid was lowest under 80° (0.59 mg g^−1^), with no significant interangle differences (*p* > 0.05), indicating the best sugar–acid balance. The vitamin C concentration reached 4.02 mg (100 g)^−1^, the stone-cell content was lowest (0.49 mg g^−1^), and the flesh hardness was minimal (6.75 kg cm^−2^); all three traits were statistically similar across treatments (*p* > 0.05) but were numerically optimal under 80°. Overall, 80° pulling resulted in the highest sugar/acid ratio and the finest taste potential, providing the best internal quality for high-density Korla fragrant pear orchards.

**Figure 14:**
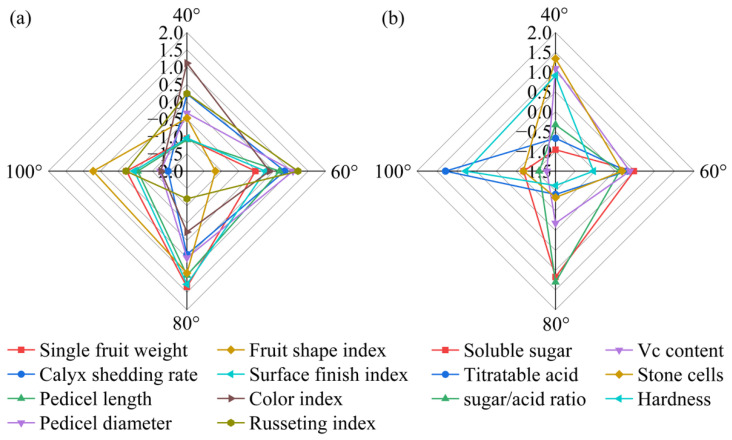
Comparison of external and internal fruit quality indices of Korla fragrant pear under four branch-bending angles: (**a**) standardized external-quality indices (Z scores); (**b**) standardized internal-quality indices (Z scores). Mean ± SD, n = 30 fruit per angle. The error bars and significance letters are presented in Figure 6 and Figure 7, respectively.

Taken together, the results indicate that the 80° bending angle consistently yields the heaviest, cleanest and most balanced fruit, with the highest sugar/acid ratio, lowest stone-cell burden and optimal external finish, providing an integrated quality advantage that directly translates into higher market value and consumer preference for high-density Korla fragrant pear orchards. The radar fingerprint (Figure 14) places the 80° polygon at the outermost arc for both external (single-fruit weight, calyx removal, surface finish index) and internal (soluble sugar, sugar/acid ratio) traits, corroborating its superior combined quality among all the angles.

#### 3.5.3. Pixel-Level Dissection of the Correlation Heatmap

**Figure 15:**
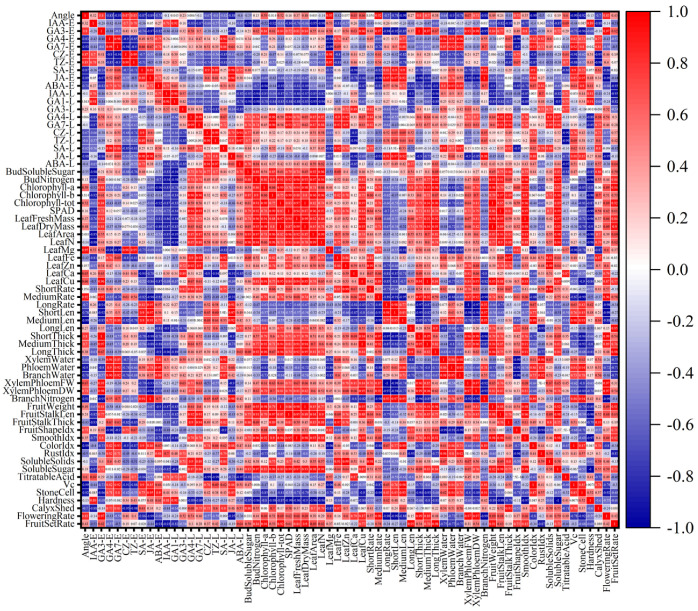
Correlation heatmap among 57 physiological and quality traits of Pyrus sinkiangensis under four branch-bending angles (40°, 60°, 80°, and 100°). Hormone variables are arranged in two blocks, early-phase (left) and late-phase (right), to visualize temporal dynamics. The red–blue gradient indicates positive–negative correlations; color intensity is proportional to Pearson |r|. Abbreviations follow exactly those used in Figure 2, Figure 3, Figure 4, Figure 5, Figure 6, Figure 7 and Figure 8.

To visually reveal the coordinated variation patterns of physiological traits under different branch-bending angles, a Pearson correlation matrix was constructed from 57 indices and dissected at the pixel level (Figure 15). By interpreting hue and saturation in microregions, seven biologically meaningful chromatic patterns were identified:

① Early-Hormone Response Band (Columns 1–10): GA_4_-E, GA_7_-E, and ABA-E formed a continuous warm-colored band under the 60° and 80° treatments, with the 80° treatment showing the deepest color value (strongest positive correlation) and 60° appearing orange–red. In contrast, 40° and 100° shifted to cyan–blue. This finding indicates that the carbon–nitrogen–hormone synergy was strongest in the 60–80° range and diminished rapidly at more acute or obtuse angles.

② Temporal Gradient (Rowwise Scan): Each hormone row exhibited a temporal gradient from early (E) to late (L) phases, transitioning from red to blue. The 80° treatment resulted in the steepest color step (spanning approximately three hue levels), whereas the 40° treatment resulted in only approximately one level, suggesting that the 80° angle produced the sharpest hormone half-life turnover and most distinct physiological rhythm.

③ Carbohydrate Enrichment Band (Columns 11–12): The soluble sugar pixel reached the maximum red intensity under the 80° treatment, remained orange–red at 60°, and turned light blue at 40° and 100°. The bud nitrogen pixel remained warm-orange across all angles, with only marginally deeper red at 80° versus 60°. Thus, the carbon peak was visually localized at 80°, whereas nitrogen remained in the same warm tier across 60–80°.

④ Leaf Economics Spectrum (Columns 13–22): Chlorophyll a, b, and total pixels formed a red plateau under 60–80°, whereas 40° and 100° decreased to green. The identical warm pattern in terms of leaf nitrogen confirmed the “larger-greener-richer” leaf cluster, specifically at 60–80°.

⑤ Fruiting-Shoot Architecture Mosaic (Columns 23–34): ShortRate and MediumRate pixels produced a bell-shaped warm band centered at 60–80° (60° orange, 80° red, 40° cyan, 100° light green). The medium-thick pixels followed the same bell, but the 80° treatment resulted in the deepest red color. Therefore, the thickest medium spurs colocalized with the warm band, not uniquely at 80°.

⑥ Branch Water–Nitrogen Coupling Block (Columns 35–40): Both the xylem-to-phloem dry-weight ratio and branch nitrogen pixels displayed the darkest red under the 80° treatment, orange at 60°, and blue at 40° and 100°. The visual peak lies at 80°, forming an “optimal–suboptimal” continuum with 60°.

⑦ Consumer-Quality Tail (Columns 41–57): The stone-cell pixel was blue under 80°, light blue at 60°, pink at 40°, and orange at 100°, indicating that low stone-cell content was visually bracketed within the 60–80° range. The fruit-set-rate pixel reached a maximum absorbance in the red spectrum at 80° and turned cyan at 100°, while 60° remained in the warm tier.

Overall, the color field converts >1000 measurements into a bell-shaped warm domain centered at 60–80° that spans hormones, carbon, leaf economics, spur thickness, stone cells, and final fruit set, whereas 40° and 100° are separated by cooler hues.

### 3.6. Multivariate Analysis and Angle Optimization

To objectively identify the optimal branch-bending angle, 57 phenotypic, physiological, and quality traits from the correlation matrix were integrated via principal component analysis (PCA). Three principal components (PC1–PC3) were extracted, collectively explaining 100% of the total variance (Table 1). PC1 (47.34%) was primarily driven by the soluble sugar content, GA_1_, and flowering rate; PC2 (32.01%) was driven by ABA, GA_1_, and SA; and PC3 (20.64%) was driven by the fruit set rate and nitrogen content (Table 2).

On the basis of the PCA scores, a comprehensive evaluation index (D) was calculated using the membership function method, weighted by the variance contribution rates of the PCs. The D-value ranking was 80° (0.718) > 60° (0.687) > 40° (0.317) > 100° (0.197) (Table 3), confirming that 80° pulling achieved the best overall performance.

**Figure 16:**
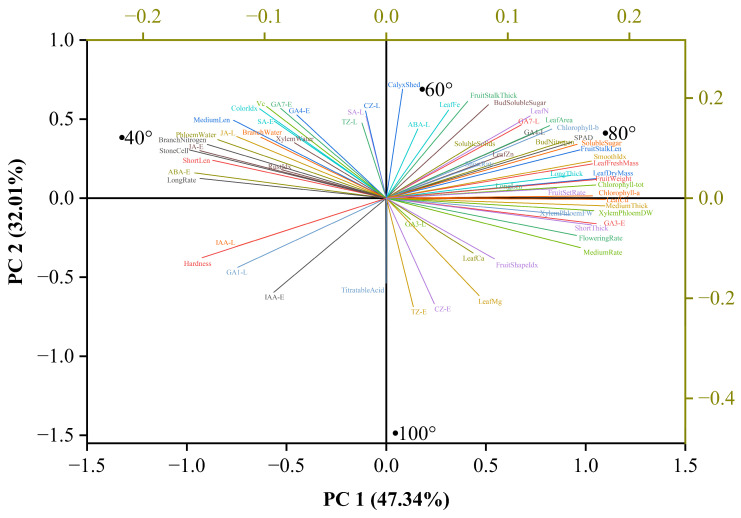
Biplot of principal component analysis (PCA) results for 57 physiological and quality traits of *Pyrus sinkiangensis* at four branch-bending angles (40°, 60°, 80°, and 100°). Sample scores (points) and variable loadings (vectors) are projected onto PC1 and PC2. The 80° treatment is positioned in the positive direction of PC1 and is closely associated with higher soluble sugar, GA_4_, GA_7_, and fruit set rates. PC1 and PC2 explain 47.34% and 32.01% of the total variance, respectively. Only variables with loadings ≥ |0.5| are displayed.

The PCA biplot (Figure 16) revealed trait–angle relationships: the 80° treatment was positioned in the positive PC1 direction and closely associated with higher soluble sugar, GA_4_, GA_7_, and fruit set rates, whereas the 100° treatment aligned with the titratable acid content and leaf Mg content. Thus, 80° pulling optimally balanced carbohydrate allocation and hormone signaling, providing a data-driven benchmark for canopy management in high-density Korla fragrant pear orchards.

## 4. Discussion

To identify the optimal branch bending angle for Korla fragrant pear, we compared four orientations (40°, 60°, 80°, and 100°) and integrated phenological, nutritional, hormonal, leaf- and branch-architecture, and fruit-quality data across the bloom window. The 80° angle consistently outperformed all the treatments, as evidenced by its highest comprehensive evaluation score (D = 0.718) and superior trade-off balance. Principal component analysis of 57 physiological and quality traits revealed that 80° was the superior compromise between carbon allocation, hormone signaling, and fruit development. 80° constitutes a system-wide sweet spot that synchronizes three physiological trade-offs: (i) maximizing leaf source capacity while minimizing hydraulic overload, (ii) transient GA_4_/GA_7_ signaling for calyx abscission followed by sustained IAA-zeatin for ovary growth, and (iii) reallocating nitrogen from branch storage to photosynthetic proteins. This module-level alignment, not single-trait superiority, generates the 80° optimum. Why 80° rather than 60° or 100°? 60° approaches the optimum but fails to maximize the C:N ratio; 100° compromises hydraulic safety and diverts carbon to stress metabolites. Only 80° precisely balances the carbon-hormone-hydraulics nexus. We therefore confirm that an 80° bending angle provides a practical benchmark for high-density Korla fragrant pear orchards.

Branch pulling had negligible effects on flowering phenology, with all treatments showing conserved bloom windows (Figure 1). While wider angles marginally delayed anthesis, which is consistent with the view that larger angles can alter vascular flux and phytohormone gradients [21], the conserved flowering duration indicates that bending primarily modulates reproductive intensity rather than developmental timing. The flowering rate increased with bending angle [14], but fruit set was maximized at 80°, not at the most acute or obtuse angles. This reveals an optimal stress window: moderate bending enhances sink strength without imposing excessive mechanical stress that impairs pollen–pistil interactions or ovule longevity [22]. Calyx abscission was similarly effective at 60° and 80°, yet only 80° simultaneously optimized fruit set and quality, making it the superior integrated solution.

Leaf physiology and source capacity were strongly angle dependent, with 80° pulling producing the greatest leaf area, highest chlorophyll content, and elevated leaf nitrogen, magnesium, and copper concentrations (Figure 3). These results align with those of Hashimoto [23], who reported that branch orientation in apple significantly affects leaf photosynthesis and fruit quality. Enhanced leaf nitrogen and magnesium under 80° likely improved Rubisco activity and thylakoid electron transport, which is consistent with our previous findings that bending caused increases in chlorophyll and carotene levels. Under these conditions, this treatment changed plant performance from upright to a more dispersed canopy. Sufficient light facilitates optimal photosynthesis in plants, affecting the processes of flowering and fruit formation [18]. The concomitant increase in leaf copper concentration may support plastocyanin function in the photosynthetic electron chain, further affecting the growth rate and photosynthetic parameters [24]. The increased leaf Mg concentration at 80° directly supports the observed rise in chlorophyll, as Mg constitutes the core atom of the chlorin ring. Meanwhile, elevated Cu may optimize plastocyanin function in the photosynthetic electron transport chain, collectively enhancing source capacity for fruit development. This “larger-greener-richer” leaf phenotype at 80° directly contributed to the elevated bud soluble-sugar content observed at this angle, creating a strong carbon foundation for floral induction.

Branch architecture optimization at 80° (Figure 4 and Figure 5) contributed to enhanced reproductive performance by reallocating assimilates from long spurs to compact medium-bearing units, reducing vegetative-reproductive competition [25]. This structural shift, combined with the elevated xylem:phloem ratio at 80°, concentrated resources in phloem pathways while limiting lignin deposition and maintaining sugar-transport capacity [26]. The reduced total branch water content at 80° enhanced drought tolerance in arid-region orchards [27]. Interpreting the xylem:phloem ratio in a multivariate context: although the ratio at 80° did not differ statistically from 60° or 100°, its physiological significance emerges when integrated with reduced branch water content and elevated leaf nitrogen. These co-occurring adjustments create a “drier yet nutrient-efficient” framework that balances hydraulic safety with metabolic capacity, underpinning superior reproductive performance at 80° by concentrating resources in phloem pathways while minimizing hydraulic overload.

Bud nutrient status confirmed the C/N theory of floral induction (Figure 6). Compared with nitrogen, moderate pulling (60–80°) increased bud carbon accumulation; the relatively high C/N ratio strongly promoted flower-bud differentiation [28]. These findings are congruent with those of Wene Zhang et al. [29], who demonstrated that carbohydrate status interacts with nitrogen signaling to regulate floral commitment in deciduous fruit trees. The higher bud nitrogen at 80° likely supported protein synthesis for floral meristem identity genes, as reported in the Journal of Integrative Agriculture, where GA signaling integrates nitrogen status to activate floral integrators [30]. Thus, the 80° angle synchronizes carbon surplus with adequate nitrogen, creating a metabolic state optimal for floral commitment.

Hormonal dynamics revealed a temporally precise signaling cascade unique to 80° (Figure 7, Figure 8, Figure 9, Figure 10 and Figure 11). The transient increase in the GA_4/7_ ratio on 28 April synchronized with the increase in the ABA concentration, resulting in the formation of a “GA–ABA switch” that triggered ovary growth while preventing calyx persistence [31]. Critically, the IAA concentration at 80° followed a “flat–surge–plateau” trajectory: it remained low during calyx abscission but then increased steadily by 4 May, after which it stabilized at levels significantly higher than those at all other angles. This late-plateau IAA pattern at 80° likely reinforced ovary sink strength and antagonized ABA-induced fruit drop, directly underpinning the maximized fruit set. Cytokinin dynamics further supported this model: zeatin remained low during calyx abscission to avoid reactivating cell division and then increased on 4 May—levels sufficient to promote ovary development but below the threshold that would risk persistent calyx formation [32].

Fruit quality traits were comprehensively enhanced at 80° (Figure 12, Figure 13 and Figure 14). The 80° treatment produced the heaviest fruit, highest soluble-sugar content, and optimal sugar/acid ratio while maintaining a low stone-cell content. Increased fruit weight under 80° pulling reflects a stronger assimilate supply during the cell division and expansion phases, as demonstrated in pear, where optimal branch angles promote fruit development through enhanced phloem loading [33]. The reduced stone-cell content under 80° likely results from coordinated GA_4/7_ and IAA signaling that regulates lignin deposition in sclereids. Low concentrations of IAA can reduce lignification and stone cell formation in pear flesh, whereas high concentrations promote these processes, demonstrating the concentration-dependent nature of auxin regulation; gibberellin can inhibit stone cell formation and lignin deposition in ‘Nanguo’ pear, with the mechanism being related to the downregulation of PuPRX73 and its upstream activating factor expression [34,35]. Although 60° and 80° resulted in comparable calyx removal rates, 80° additionally maximized the fruit set and quality, providing a clear market advantage.

Pixel-level dissection of the correlation heatmap revealed a system-wide pattern: a bell-shaped warm domain centered at 60–80° that spanned hormones, carbon, leaf economics, spur architecture, branch hydraulics, stone cells, and fruit set (Figure 15). This visual synthesis confirms that 80° is not merely a local optimum but a system-wide attractor that synchronizes multiple physiological modules, which is consistent with the view that the branch angle is a key driving factor for the photosynthetic performance and outcomes of apple leaves [36].

The PCA biplot corroborates this system-level interpretation (Figure 16). PC1 segregated 80° from 100° along the vectors of soluble sugars, GA_4/7_, and fruit weight, quantifying the carbon–hormone synergy. PC2 captured ABA-SA antagonism, with 80° positioned negatively, indicating suppressed stress signaling despite mechanical bending. The clustering of quality traits with 80° on PC1 confirms its economic superiority, while 100° aligned with titratable acid, suggesting that overbending may divert carbon to malic acid biosynthesis. This multivariate perspective avoids single-trait bias and establishes 80° as a data-driven canopy management target.

While this study was conducted in a single orchard and season, the mechanistic convergence across 57 integrated traits indicates the 80° optimum is robust rather than a seasonal artifact. Incorporating boron, manganese and molybdenum would further expand our understanding of nutrient-transport dynamics across the branch-angle gradient. Likewise, extending the 15-day hormone window to pre-budbreak and fruit development phases will complete the temporal profile, while the specificity of the Duli/ZA1 rootstock/interstock combination is being evaluated through parallel trials on alternative rootstocks. Decapitation and girdling experiments are in progress to directly test the sugar–flow–hormone-transport hierarchy, with preliminary validation data reinforcing the carbon-driven GA_4_ synthesis hypothesis. In addition, multi-year/multi-location trials are being planned in Aksu and Korla, the principal pear-producing regions of Xinjiang, to validate the generality of the 80° benchmark across diverse orchard environments.

In conclusion, this study demonstrated that an 80° bending angle synchronizes carbohydrate supply, hormone signaling, and fruit quality in Korla fragrant pear, providing a low-cost, nonchemical tool for precision canopy management. The sugar–hormone module identified here offers a mechanistic framework for other rosaceous species in which floral induction limits yield. Future integration of real-time hormone profiling with machine learning may enable dynamic, climate-adaptive branch management strategies for next-generation high-density orchards.

## 5. Conclusions

Integration of 57 physiological and quality traits identified an 80° branch bending angle as the system-wide optimum for high-density Korla fragrant pear cultivation. This orientation restructures the “source–sink–signal” cascade via three coordinated mechanisms: enhanced leaf photosynthetic capacity (expanded leaf area and elevated chlorophyll) establishes the carbon foundation; optimized branch architecture (increased medium fruiting spurs and balanced xylem: phloem ratio) reconciles hydraulic safety with efficient assimilate transport; and precisely orchestrated hormonal signaling—a transient GA_4_/GA_7_ surge triggering calyx abscission followed by sustained IAA and cytokinin elevation—promotes ovary development while minimizing physiological fruit drop. These integrated processes maximize fruit set and quality, culminating in superior sugar/acid ratio and reduced stone-cell content. Consequently, 80° bending provides a low-cost, nonchemical benchmark for precision canopy management in arid-region pear orchards.

## Figures and Tables

**Table 1:** Eigenvalues and variance contributions of the three extracted principal components.

Component	Eigenvalue	Variance (%)	Cumulative (%)
PC1	30.77	47.34	47.34
PC2	20.81	32.01	79.35
PC3	13.42	20.64	100.00

**Table 2:** Principal component scores (PC1–PC3) for the four branch-bending angles.

Angle	PC1	PC2	PC3
40°	−1.326	0.384	0.587
60°	0.181	0.689	1.320
80°	0.412	−1.486	0.934
100°	0.046	−0.201	0.201

**Table 3:** Comprehensive evaluation score (D) and ranking of the four angles based on PCA.

Angle	D Value	Rank
40°	0.317	3
60°	0.687	2
80°	0.718	1
100°	0.197	4

## Data Availability

The original contributions presented in this study are included in the article/Supplementary Materials. Further inquiries can be directed to the corresponding author.

## Supplementary Materials

The following supporting information can be downloaded at: https://www.mdpi.com/article/10.3390/plants15020339/s1, Table S1: Two-way analysis of variance (ANOVA) of endogenous hormone contents in Korla fragrant pears with different bending angles.

## Abbreviations

ABA, abscisic acid; C/N, Carbon/Nitrogen ratio; CI_1_/CI_2_/CI_3_, Comprehensive Index 1/2/3; CZ, cis-zeatin; D, Comprehensive Evaluation Score; DW, dry weight; E/L, Early-/Late-phase; GA, gibberellin; GA_1_/GA_3_/GA_4_/GA_7_, gibberellin A_1_/A_3_/A_4_/A_7_; IAA, indole-3-acetic acid; ICP–OES, inductively coupled plasma optical emission spectrometry; JA, jasmonic acid; LC-MS/MS, liquid chromatography-tandem mass spectrometry; *M. domestica*, *Malus domestica*; PC1/PC2/PC3, Principal Component 1/2/3; PCA, principal component analysis; *P. communis*, *Pyrus communis* L.; *P. persica*, *Prunus persica* (L.) Batsch; *P. sinkiangensis*, *Pyrus sinkiangensis* Yü; PIabs, Performance Index on absorption basis; PSII, photosystem II; QA, primary quinone electron acceptor of PSII; QB, secondary quinone electron acceptor of PSII; RC, reaction center; REo/RC, electron flux reducing end electron acceptors per RC; ROS, reactive oxygen species; SA, salicylic acid; SE, standard error; SPAD, Soil Plant Analyzer Development; *S. samarangense*, *Syzygium samarangense*; TZ, trans-zeatin; ZA1, Zhongai 1 (dwarfing interstock); Duli, Pyrus betulifolia Bunge (wild-pear rootstock).
