# Supplementary material for: Optimal Branch Bending Angle for Korla Fragrant Pear: A Multi-Trait Physiological Trade-Off Framework
Source: Plants (Basel). 2026 Jan 22;15(2):339. doi: 10.3390/plants15020339 (PMC12845164; doi:10.3390/plants15020339)
Supplement: Supplementary file 1 [file plants-15-00339-s001.zip › plants-4090649-supplementary.pdf]

plants

MDPI

Supplementary Material

Table S1. Two-way analysis of variance (ANOVA) of endogenous hormone contents in Korla fragrant pears with different bending angles.

Factor	IAA	ABA	GA_1	GA_3	GA_4	GA_7	SA	JA	CZ	TZ
Period	2024.57**	114517.50**	738.66**	23.88**	1288.86**	342.93**	351.80**	11806.06**	152.29**	80.72**
Branching angle	303.41**	2762.41**	9.75**	4.74	76.21**	15.81**	139.40**	1486.89**	36.08**	19.25**
Period×Branching angle	721.11**	4381.86**	19.92**	6.74**	190.27**	83.77**	31.68**	2745.80**	39.92**	21.09**

Note: F values from two-way ANOVA with the factors Time, Angle and their interaction. Asterisks indicate significance levels: *P < 0.05, **P < 0.01, ***P < 0.001. n = 4 sampling dates × 3 biological replicates per angle.

Practical Implications: The 80° bending angle offers a low-cost, nonchemical precision management tool that is particularly suitable for high-density orchards in arid regions. By synchronizing carbohydrate allocation with hormonal signaling, this strategy addresses fruit set variability and calyx persistence in Korla fragrant pear systems, potentially replacing exogenous growth regulators and reducing manual labor.

Future Directions: Cross-cultivar validation of the universality of this module is needed, and climate-adaptive dynamic management strategies integrating real-time hormone monitoring with machine learning should be explored. This study establishes a methodological foundation for data-driven modern orchard management.
